# The Impact of Phenotypic Switching on Glioblastoma Growth and Invasion

**DOI:** 10.1371/journal.pcbi.1002556

**Published:** 2012-06-14

**Authors:** Philip Gerlee, Sven Nelander

**Affiliations:** 1 Sahlgrenska Cancer Center, Institute of Medicine, Göteborg, Sweden; 2 Mathematical Sciences, University of Gothenburg and Chalmers University of Technology, Göteborg, Sweden; University of Notre Dame, United States of America

## Abstract

The brain tumour glioblastoma is characterised by diffuse and infiltrative growth into surrounding brain tissue. At the macroscopic level, the progression speed of a glioblastoma tumour is determined by two key factors: the cell proliferation rate and the cell migration speed. At the microscopic level, however, proliferation and migration appear to be mutually exclusive phenotypes, as indicated by recent *in vivo* imaging data. Here, we develop a mathematical model to analyse how the phenotypic switching between proliferative and migratory states of individual cells affects the macroscopic growth of the tumour. For this, we propose an individual-based stochastic model in which glioblastoma cells are either in a proliferative state, where they are stationary and divide, or in motile state in which they are subject to random motion. From the model we derive a continuum approximation in the form of two coupled reaction-diffusion equations, which exhibit travelling wave solutions whose speed of invasion depends on the model parameters. We propose a simple analytical method to predict progression rate from the cell-specific parameters and demonstrate that optimal glioblastoma growth depends on a non-trivial trade-off between the phenotypic switching rates. By linking cellular properties to an *in vivo* outcome, the model should be applicable to designing relevant cell screens for glioblastoma and cytometry-based patient prognostics.

## Author Summary

In this work, we develop a spatial mathematical model in order to analyse the growth behavior of the brain tumour glioblastoma. Tumours of this type have a diffuse boundary, with considerable local invasion of surrounding brain tissue, making surgery difficult. At the cellular level, the progression of a glioblastoma is known to depend on the balance between cell division (proliferation) and cell movement (migration). Based on recent evidence, our model assumes that each cell in a glioblastoma tumour resides in either of two mutually exclusive states: proliferating or migrating. From a probabilistic model of switching between these two phenotypes, we go on to derive equations that link cellular phenotypes to disease progression. The model has several possible applications. For instance, it could be used to predict the rate of disease progression in an individual patient, and to improve screening methods.

## Introduction

Cancer progression is the macroscopic outcome of numerous cellular processes, such as elevated proliferation rates, defects in apoptosis regulation and abnormal angiogenesis [1]. In the development of targeted anticancer therapies, the proliferation, survival and angiogenesis phenotypes are often singled out as the most important. Recently, however, much attention has been given to cancer cell *migration* as a possible therapeutic target, since it underlies both the local invasive process whereby cancer cells degrade and move through the adjacent tissue, and the formation of distant metastases.

The importance of cancer cell migration is perhaps most evident in the common brain tumour glioblastoma, which is characterised by rapid and infiltrative growth into the surrounding brain tissue. In glioblastomas, neoplastic cells are often found at a long distance (several centimeters) from the main tumour mass. This diffuse growth pattern presents a difficult clinical problem, since residual ‘satellite cells’ can mediate rapid recurrence of the disease after surgery [2]. Key factors that underlie glioblastoma cell invasiveness include high migration speeds in comparison to other types of cancer (up to 100 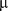
m/h) and the fact that brain parenchyma provides a penetrable substrate for invasion [3]. Thus, inhibition of migration pathways might constitute an interesting complement to standard glioblastoma therapies that seek to inhibit cell proliferation rate or angiogenesis. Several pathways have been suggested to mediate the highly migratory phenotype of glioblastoma cells, including signaling via Focal adhesion kinase (FAK) [4], Phosphoinositide 3-kinase PI3K [5] and Signal transducer and activator of transcription 3 (STAT3) [6]. Other concepts for targeting of migration have also been proposed, including inhibition of integrins [7], perturbing the interactions between ECM components [8], and administering lithium chloride [9]. Further, potential gene targets have also been revealed using molecular profiling efforts [10].

However, the potential of migration as a therapeutic target is complicated by the strong dependency between migration and proliferation phenotypes. Early *in vitro* experiments by Giese et al. [11] showed that when plated on a substrate, that supports migration, the proliferation rate of glioblastoma cells is markedly reduced. Later, it was shown that cells at the tumour's invasive rim proliferate more slowly than cells in the central parts of the tumour, again suggesting that migration has a ‘cost’ in terms of reduced proliferation [2]. These and several additional observations led to the so called ‘go or grow’ hypothesis, stating that migration and proliferation are mutually exclusive phenotypes. Although a molecular explanation for this dichotomy still is missing, it has been suggested that cytoskeleton dynamics could be limiting, as it is involved in both cell division and force generation in migration [12].

In recent experiments, *in vivo* imaging of fluorescent glioblastoma cells enabled direct observation of phenotypic switching between the ‘go’ state (migration) and the ‘grow’ state (proliferation). More precisely it was observed that glioma cells move in a saltatory fashion, where bursts of movement are interspersed by periods of immobility, and it is during these stationary periods that the cells divide [13], [14]. Taking these observations into account allows for a more comprehensive understanding of glioblastoma progression, where tissue-level traits, such as progression rate, emerge from cell-level behaviour. Mathematical models at the resolution of individual cells enable a quantitative connection between these scales, and can hence be of great assistance.

Here, we focus on the relationship between cell-level phenotypic switching in glioblastoma, and the properties of the tumour as a whole. In particular we elucidate how the growth rate of the tumour and speed of invasion depends on the specific underlying microscopic parameters, such as phenotypic switching rates, rate of apoptosis *et cetera*. Please note that the we use the word ‘invasion’ to denote the process by which glioma cells spread into and displace the surrounding brain tissue, and do not refer to branched finger-like growth patterns. Although several models of glioma growth have previously been proposed (see next section), this model is the first to connect experimentally measurable cell-level traits with gross tumour volume in an analytical way. This yields hope for the future understanding of glioma biology and therapy, since it is the understanding of how drug induced changes on the cell-level scale propagate to the organ scale, that are required in order to accurately predict therapeutic outcome.

In the following we first review previous work in the field of glioblastoma modelling and then proceed by introducing our individual-based (IB) stochastic model of glioma growth. From this model we derive an approximate continuum description of the system, whose properties are compared to the IB-model. We proceed to analyse the continuum model to reveal the influence of the model parameters on the rate of spread of the tumour, and finally discuss our results in the context of other models and experimental results.

### Previous work

The growth of glioblastomas was first modelled by means of a continuum approach, which captures the two main features of glioma cells: proliferation and migration ([15], [16], and [17], chapter 11). In that model, the partial differential equation (PDE) that describes the time evolution of the concentration of glioma cells 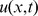 in space and time has the form:
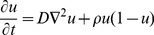
(1)where the migration is captured by a diffusion term with diffusion coefficient 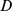 (first term) and proliferation of the glioma cells is described by a locally logistic growth function with growth rate 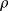 (second term). This equation is known as the Fisher (or Kolomogorov) equation, and was first derived in order to describe the spread of an advantageous gene in a spatially extended population [18], [19]. The derivation, originally by Fisher and later refined by Kolmogorov, starts by assuming a contact distribution that describes the probability of migration between two spatial locations, and by then assuming that all moments of that distribution higher than two are negligible (known as the diffusion approximation, see for example [20]) one arrives at the above equation.

The Fisher equation has been of particular interest since it gives rise to traveling wave solution 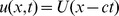, whose shape is preserved and position in space is shifted at a speed 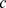 as time progresses. Significant interest has been devoted to determining the wave speed 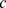, and it has been shown that for reasonable initial conditions (exponentially decaying [20] or of compact support [19]) the wave speed is given by 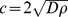 [21]. The speed of propagation thus depends on both the motility, captured in the diffusion constant 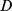, and the rate of proliferation 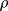. Both 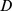 and 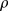 can be determined from time-course Magnetic Resonance Imaging (MRI) data from actual patients, and it has been shown that their values are of prognostic power [22].

The above modeling approach rests on the assumption that glioblastoma cells follow a random walk (which at the macroscopic scale corresponds to the diffusion of cells). Recently this assumption has been under scrutiny, and this has led to a number of explorations of non-random migration, i.e. where migration is influenced by biological processes such as cell-cell signaling, oxygen pressure, nutrient availability and phenotype switching. In one line of work, Aubert et al. [23] used an individual based (IB) model to show that attraction between glioblastoma cells is likely to influence the dynamics of tumour invasion. Deroulers et al. [24], derived the macroscopic PDE for this case, obtaining a density dependent diffusion equation (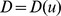 in terms of eq. (1)), whose solution deviates significantly from the Fisher-Kolmogorov PDE (see also [25], [26]). Khain et al. [27], used IB models to characterise the role of hypoxia in glioblastoma, showing that reduced oxygen levels may down-regulate cell-cell adhesion, leading to increased motility.

The cellular behaviour implied in the ‘go-or-grow’ hypothesis (see Introduction) is also thought to affect migration and growth dynamics of glioblastomas, in a manner that is not captured by the Fisher-Kolmogorov equation. Hatzikirou et al. [28] proposed a lattice-gas cellular automaton model in which the switching between the proliferative (P) state and migratory (M) state is driven by lack of oxygen, and went on to show that in the corresponding macroscopic (Fisher) equation, there is a tradeoff between diffusion and proliferation reflecting the inability of cells to migrate and proliferate simultaneously. Similar results where obtained by Fedotov and Iomin [29] but with a different type of model known as continuous time random walk model. That model contains two distinct subpopulations (P-cells which are stationary and divide and M-cells that perform random walks), and a cell switches from one compartment to the other after a time 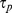 (and 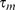 respectively) which is exponentially distributed. They analytically show that the spreading rate is smaller than one would expect from the Fisher equation (1). Finally, Lewis and Schmitz have studied the general relation between organism migration and proliferation and its impact on population spread using reaction-diffusion equations [30]. They show that the system exhibits travelling wave solutions and that the wave speed depends on the rates of switching between the states.

The model that we propose draws from these previous models, but is different in some crucial ways. We consider two distinct subpopulations with a stochastic switching in between (as in Fedotov and Iomin, and Lewis and Schmitz), but instead of starting with a continuum description, we begin with an IB-model in which the cells occupy a lattice and obey size exclusion (as in Deroulers et al.), and from that derive a system of PDEs. This allows for an analytical treatment of the IB-model which establishes a connection between cell characteristics and the macroscopic behaviour of the system previously not demonstrated.

## Results

### The individual-based stochastic model

The cells are assumed to occupy a 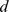-dimensional lattice (we will consider 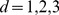), containing 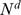 lattice sites, where 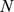 is the linear size of the lattice and each lattice site either is empty or holds a single glioma cell. This means that we disregard the effects of the surrounding brain tissue, such as the different properties of grey vs. white matter [31], and the presence of capillaries which might influence the behaviour of the cancer cells. But since the soft tissue in the brain presents little resistance to invading cancer cells and the precise nature of interaction with stromal cells is unclear, focusing on the dynamics of the glioma cells is a reasonable first approximation. Further, the process of angiogenesis, which has been modelled extensively [32], is ignored, and we hence assume the growing tumour to be well vascularised. For the sake of simplicity we do not consider any interactions between the cancer cells (adhesion or repulsion), although this could easily be included, and we also disregard other types of mechanical interactions, such as cell pushing (see Discussion).

The lack of knowledge of the intra-cellular dynamics and extra-cellular cues that lead to the phenotypic switching behaviour poses a problem, but we will circumvent it by, as a first approximation, considering the switching as a stochastic event. The behaviour of each cell is therefore modelled as a time continuous Markov process where each transition or action occurs with a certain rate, which only depends on the current and not previous states, known as the Markov property. The rates are interpreted in the standard way, i.e. if transition in a variable 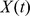 from state 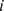 to 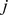 occurs at rate 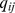 then the probability of a transition occurring in the time interval 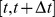 is given by
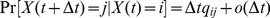
(2)where 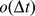 means that remaining terms are bounded from above by 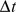, and thus that in the limit of small 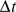 the transition probability is proportional to 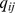.

Each cell is assumed to be in either of two states: proliferating or migrating, and switching between the states occurs at rates 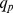 (into the P-state) and 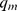 (into the M-state). A proliferating cell is stationary, passes through the cell cycle, and thus divides at a rate 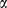. The daughter cell is placed with uniform probability in one of the empty 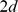 neighbouring lattice sites (using a von Neumann neighbourhood). If the cell has no empty neighbours cell division fails. A migrating cell performs a size exclusion random walk, where each jump occurs with rate 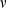. Size exclusion means that the cell can only move into lattice sites which were previously empty. If only a motile subpopulation is considered, size exclusion does not affect the macroscopic diffusive nature of a population of random walkers, but if two or more subpopulations are taken into account then, as we shall later see, diffusion becomes non-linear. Lastly, cells are assumed to die, through apoptosis, at a rate 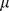 independent of the cell state. Since this type of cell death is associated with cell shrinking and rapid removal of the dead cell, a cell which goes through apoptosis is instantly removed from the lattice and leaves an empty lattice site behind.

**Figure 1:**
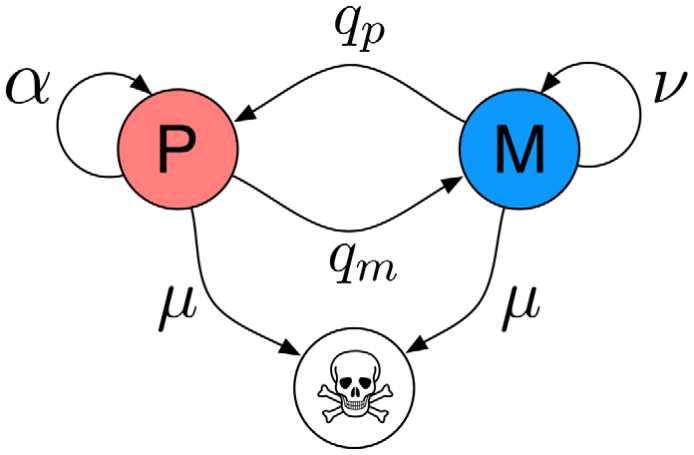
Schematic describing the continuous time Markov process each cell is subject to. A living glioma cell can be in either of two states, proliferating (P) or migrating (M), and transitions between the states with rates 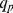 and 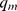 respectively. A P-cell divides at rate 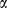 while an M-cell moves with rate 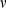. Both cell types go into apoptosis and die with a constant rate 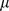.

The stochastic process is depicted schematically in figure 1. In fact the whole system comprises a continuous time Markov chain with a finite, but very large state space, containing 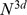 different states, where 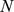 is the linear size of the system and the 3 comes from the three distinct lattice states: empty, P-cell and M-cell.

### Parameters

We will consider a lattice of linear size 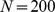 with a spacing of 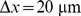, the typical size of a cancer cell. For the most part we will consider the system in 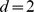 dimensions, which means that we simulate a lattice, which corresponds to a 4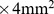 slice of tissue. This is of course considerably smaller than a clinically relevant glioma, but sufficient to capture the effects of the phenotypic switching on tumour growth rate. The time scale of the model is set to agree with that of the cell cycle (approximately 24 hrs [2]) which means that the proliferation rate 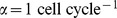, and that we scale all other parameters accordingly. We are mainly interested in the effect of the phenotypic switching rates 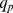 and 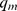 on the growth of the tumour and they will therefore be varied within a biologically reasonable range. It follows from equation (2) that the time spent in one phenotypic state is exponentially distributed with parameter 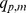 and thus that the average time spent in each state is given by 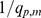 (cell cycles). It has been observed that the switching from a stationary to motile state (and back) does not occur faster than on the time scale of one hour [13]. This gives an upper limit on the transition rates, which since time in the model is measured in cell cycles, is given by 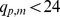.

The motility rate is set to 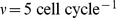. This means that a motile cell on average moves one lattice site, i.e. 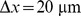, in a time 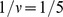 cell cycles, which gives a linear velocity of 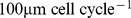, that lies within experimentally determined values of 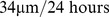 [14] and 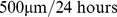 [13]. The rate of apoptosis is set to the value 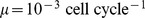, which is small compared to the other transition rates in the model.

### Simulations

Our concern is the influence of the microscopic cell-level parameters on the growth rate of the tumour as a whole, and we will therefore measure the size the tumour after a fixed time for a given set of initial conditions, as a function of the phenotypic switching rates. More specifically we will measure the tumour mass (the total number of cells), and also later, quantify the rate of spread by measuring the velocity of the tumour interface. The precise initial condition of the model has little impact on the long-term rate of spread (data not shown), but in line with the clonal origin of cancer we initialise the model with a single cell (in the P-state). All simulations of the IB-model are carried out using the commonly employed Gillespie algorithm [33].

**Figure 2:**
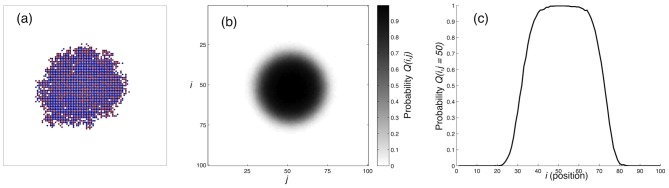
Simulating the individual-based model. Simulation results for 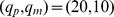 and 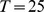. (a) The result of a single realisation where P-cells are coloured blue and M-cells are coloured red. (b) The occupancy probability 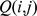 of finding a cell at location 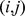 obtained by averaging over a large number of simulations. (c) A slice through the function in panel (b) at 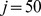.

Figure 2 illustrates the results of simulating the model in two dimensions for 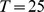 cell cycles when 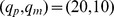 in three different ways. Panel (a) shows the result of a single simulation, where P-cells are coloured blue and M-cells are red, (b) shows the results of the model averaged across a large number of realisations and gives the occupancy probability 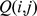 of finding a cell at location 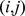 on the lattice, and (c) shows a slice through this function 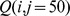. This figure gives us a general idea of the growth dynamics of the model. The tumour grows with a radial symmetry, and exhibits a solid core, while the tumour margin is diffuse and somewhat rugged. Please note that the time span considered in this simulation is smaller than the time scale of actual glioblastoma growth, which usually occurs on the time scale of months to years, but still sufficient to investigate the dynamics of the model.

**Figure 3:**
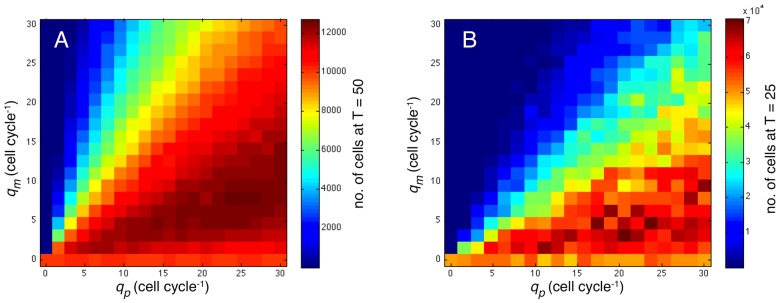
The impact of phenotypic switching rates on tumour mass. (a) The tumour mass at 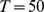 for the 2-dimensional model as a function of the phenotypic switching rates 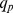 (the rate at which cells become proliferative) and 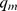 (the rate at which they become motile). (b) The tumour mass at 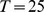 for the 3-dimensional as a function of 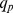 and 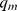. The results in 2 and 3 dimensions are similar, although a larger variability seems to exists in the 3-dimensional case.

In order to quantify the dependence on the phenotypic switching rates we measured the tumour mass at 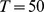 in the parameter range 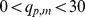. The results are displayed in figure 3a and show a strong dependence on the two parameters. For 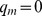 all cells are in the proliferative state, and as expected the mass is independent of 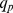. The other extreme where 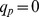 gives rise to tumours with a zero mass, which occurs since the motile cells cannot multiply and eventually die off due to the small but non-zero apoptosis rate 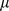. These results are intuitive, but what is more interesting is that tumour cells with intermediate switching rates are the ones that give rise to the largest tumours. Although migratory behaviour does not directly contribute to an increase in the number of cancer cells it has the secondary effect of freeing up space which accelerates growth compared to the tumours dominated purely by proliferation (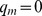). The results suggest that for each 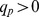 there is a 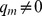 which gives a maximal tumour growth rate. These results also hold for the more biologically plausible 3-dimensional case (see figure 3b). Although the maximal tumour mass seems to occur for a smaller 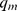, and the region of parameter space giving rise to small tumours is considerably larger (upper left region), the qualitative behaviour is similar. The implications of the observation that 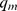 influences tumour size in a non-monotone way will be discussed later, and we will now proceed to an analytical treatment of the problem.

### Derivation of continuum model

In an effort to get a deeper understanding of the somewhat unintuitive relationship between tumour growth rate and phenotypic switching rates we will derive a set of two coupled PDEs which will serve as an approximate way of describing the time evolution of the occupancy probability 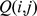 (see figure 1b and c). For the sake of clarity we will however constrain the derivation to a one-dimensional system. In fact, radially symmetric travelling wave solutions with constant velocity do not exist for 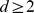, but instead the velocity of the front depends on the local curvature. However, for large enough times the interface of the circular (spherical) solution has almost zero curvature and its dynamics is well approximated by the one dimensional solution. A further simplification in our derivation is that we assume the occupancy probabilities of neighbouring sites as independent, which in practice means that we for example allow ourselves to write: 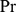
(site 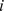 empty and site 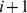 occupied) = 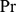
(site 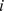 empty)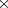 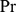
(site 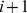 occupied).

The derivation is carried out in two steps: firstly, a set of coupled master equations, for the two sub-populations, are derived by considering the processes which alter the occupation probabilities at a given site, and secondly these master equations are approximated by a set of PDEs. In brief, the second step is achieved by identifying the on-lattice master equations with a set of coupled PDEs, which when discretised on the length scale of the lattice spacing, equal the master equations. The full derivation is given in Methods and results in the following system of coupled PDEs:
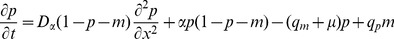
(3)

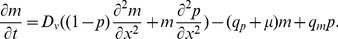
(4)Here 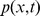 denotes the density of proliferating cells, and 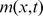 that of the motile cells. In equation (3) we recognise the first term as a diffusion term, modulated by a density-dependent prefactor and the second term as a logistic growth term. The remaining terms are due to the switching between the subpopulations and to apoptosis. In the equation for the motile cells (4) there is also density-dependent diffusion, but of a different type. This is typical of a two species size exclusion process [34], and contains the second-derivative of both species. The values of the diffusion constants are 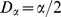 and 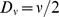, and depend crucially on the choice of spatial scale, which for simplicity is chosen to be that of the cell size 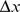. If a coarser spatial scale is considered then the diffusions constants would have to be scaled accordingly (see Methods for details). We will now proceed to investigating the properties of this system of PDEs through both numerical solutions and analysis.

### Travelling wave solutions and their velocity

The first question one might ask about a system of equations that presumably describes tumour growth is if it exhibits tumour invasion and hence travelling wave solutions, and further how the model parameters influence the wave speed, i.e. the velocity of the invading tumour front. The results from the IB-model (figure 2 and 3) suggest that the switching rates 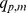 strongly influence the tumour mass, and hence we expect them to also have an effect on the speed of invasion.

In order to investigate this, we first solved the continuum model (3)–(4) numerically (which actually corresponds to reverting to the master equations eq. (8)–(9)), for a range of parameter values, in the domain 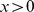. The initial condition was set to 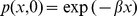, 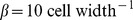 and 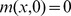, meant to represent a situation where a tumour is initiated by a small number of proliferating cells (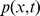) and no migratory cells (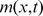). In fact the balance between 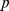 and 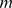 in the initial condition is largely irrelevant for the long-term dynamics of the model, the exceptions being the extremes 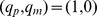 and 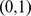, when flow between the phenotypes is unidirectional or completely blocked. The boundary conditions of the domain were set to no-flux.

**Figure 4:**
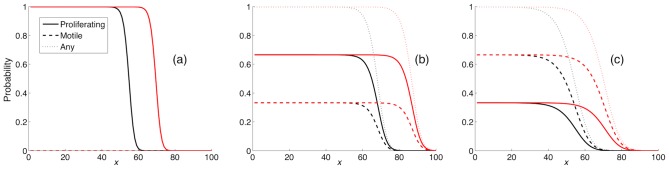
Numerical solutions of the continuum model. Solutions of the 1-dimensional continuum model (equation (3) and (4)) for three different values of the switching rates at 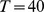 (black) and 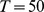 (red). The initial condition was set to 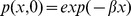, 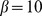 and 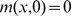. In (a) 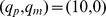, (b) 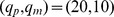 and in (c) 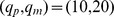. All solutions exhibit similar characteristics with an invading front of cancer cells stretching into the healthy tissue, similar to the solutions of the Fisher equation (1). The similarity between the solutions at the two different time points clearly shows that our system exhibits travelling wave solutions.

The results can be seen in figure 4 and shows the occupancy probabilities after 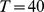 and 50 cell cycles. From these results it is clear that the system exhibits an invading front of cancer cells, similar to what is observed for the Fisher equation. The leftmost panel (a) shows the dynamics of a tumour which only contains proliferating cells, while (b) and (c) exhibit a mix of P- and M-cells. The solutions remain stationary in a moving frame, suggesting that travelling wave solutions exist, with wave speeds 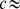
1.48, 1.88 and 1.63 respectively. These numerical results mirror what was seen in figure 2, where an intermediate 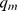 gave rise to the largest tumours. Please note that the wave speed for the case 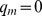 is roughly what one would expect from a Fisher equation with 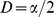 and 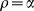, since 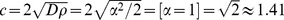. However, this is not what occurs in the IB-model where the tumour interface moves at an average velocity of 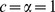. The source of this discrepancy is the assumption of independence between sites, which applies the least in this particular case (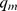 = 0), when there is no random motion within the cell population. Migration of the cells tends to break up the correlations that build up as the tumour is growing, and as we later shall see, the continuum approximation works better when the cells are more motile.

The observation that the numerical solutions are stationary in a moving frame suggests the existence of travelling wave solutions. In order to close in on these solutions, and get an estimate of their velocity, we will make use of the travelling wave ansatz: 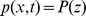 and 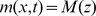 with 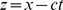, where 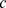 is the velocity of the interface. The problem of determining how 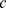 depends on the model parameters is solved by applying phase-space analysis (see Methods), and boils down to a four-dimensional eigenvalue problem, namely to find the smallest 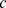 such that the eigenvalues of the Jacobian all have imaginary part equal to zero. This problem is analytically intractable, but provides us with a numerically easy way of determining the velocity.

### Influence of the parameters on the wave speed

Although phase-space analysis does not yield an analytic closed-form expression for the wave speed 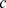, it still provides us with a computationally simple way of determining the velocity of the tumour margin in the model: for a given set of parameter values we start by setting 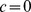 and calculate the eigenvalues of the Jacobian (18) (or equivalently the roots of the corresponding characteristic polynomial 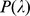). If not all eigenvalues are real we increment 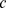 slightly and reevaluate the eigenvalues. This procedure is terminated as soon as we find all eigenvalues real, and the value of 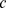 for which this occurs corresponds to the wave speed for those parameter values.

**Figure 5:**
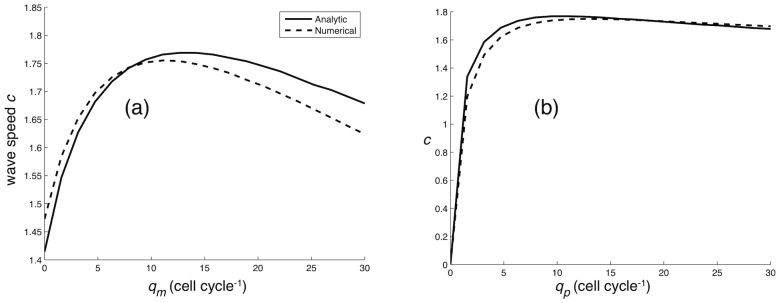
Comparison between continuum model and analytical result. The wave speed of the propagating tumour margin determined from both phase space analysis (solid line) and numerical simulation (dashed line). In (a) the switch rate to proliferation is fixed at 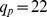, while in (b) we have fixed 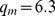.

In order to test the validity of the wave speed analysis we compared the wave speeds obtained in the continuum and IB models with those from the phase space analysis. For the continuum model an estimate of the wave speed was obtained by, from the initial condition 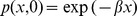 (for proliferating cells), 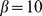 and 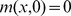 (for migrating cells), integrating the equations (3)–(4) for 200 time steps (cell cycles). From these solutions we estimated the velocity of the front by measuring the position of a reference point 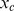, defined as the point where 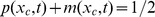, as a function of time. The comparison between the speed of propagation in the numerical solution and the wave speed obtained from the phase space analysis is shown in figure 5. The agreement is fairly good and the discrepancies are probably due to error in integration and the deviation in the numerical solution from a perfect travelling wave, which from a given set of initial conditions, is only attained in the limit 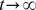. However, since we are interested in biologically relevant scenarios the time frame considered is reasonable.

**Figure 6:**
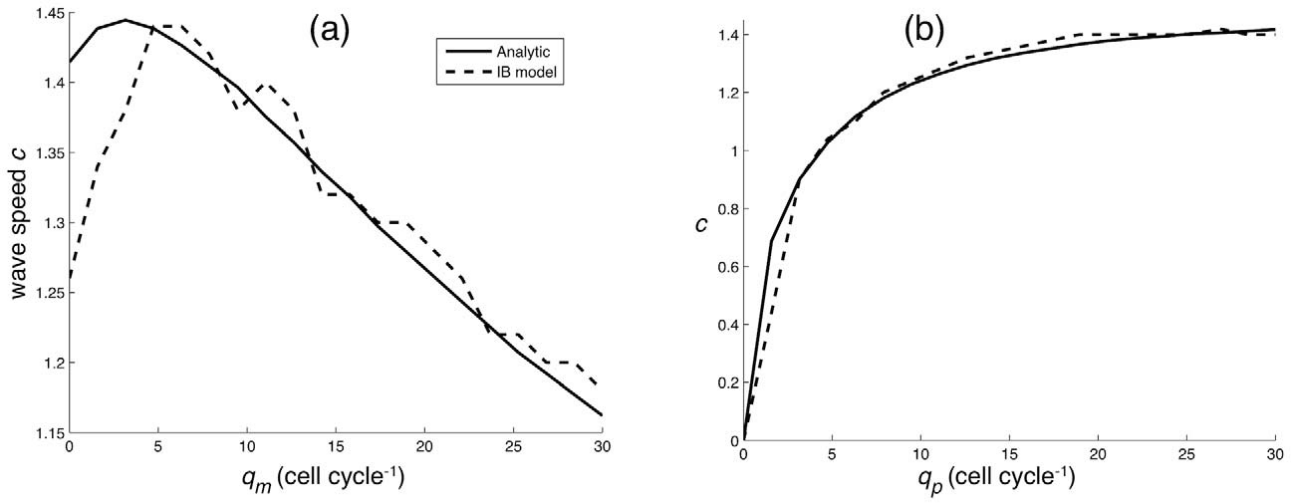
Comparison between IB-model and analytical result. The wave speed of the propagating tumour margin determined from both the individual-based model (dashed line) and phase space analysis of the continuum approximation (solid line). In (a) the switch rate to proliferation is fixed at 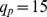, while in (b) we have fixed 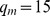.

When it comes to the IB model, we have to take into account the stochastic nature of the model, and therefore need to estimate the average margin velocity from a large number of simulations (100 independent realisations). Each simulation was started with a single P-cell at the center of the lattice and the model was simulated for 100 time steps (cell cycles). In each time step the location of the cells was recorded and from this we calculated the occupation probability 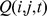 of finding any cell at location 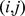 at time 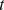. The wave speed was then approximated by taking the average propagation speed of 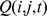 in the 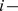 and 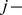
direction (as in the PDE case). In comparing with the two-dimensional simulations we need to rescale the diffusion coefficient 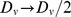, since cell movement occurring tangential to the two-dimensional front does not contribute to its propagation. The result can be seen in figure 6, which shows that the analytical result is in good agreement with the discrete individual-based model. The disparity between the IB-model and the analytic answer is largest for small 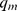, when the dynamics are dominated by proliferation. This is to be expected since for larger 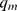 the movement of the cells decorrelates the sites, and hence our assumption about site independence is closer to truth. The analytical results recapitulates the non-monotone dependence on 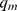 and using this method we found that the largest tumours occur when 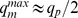, i.e. when the ratio between the switching rates is 1∶2.

**Figure 7:**
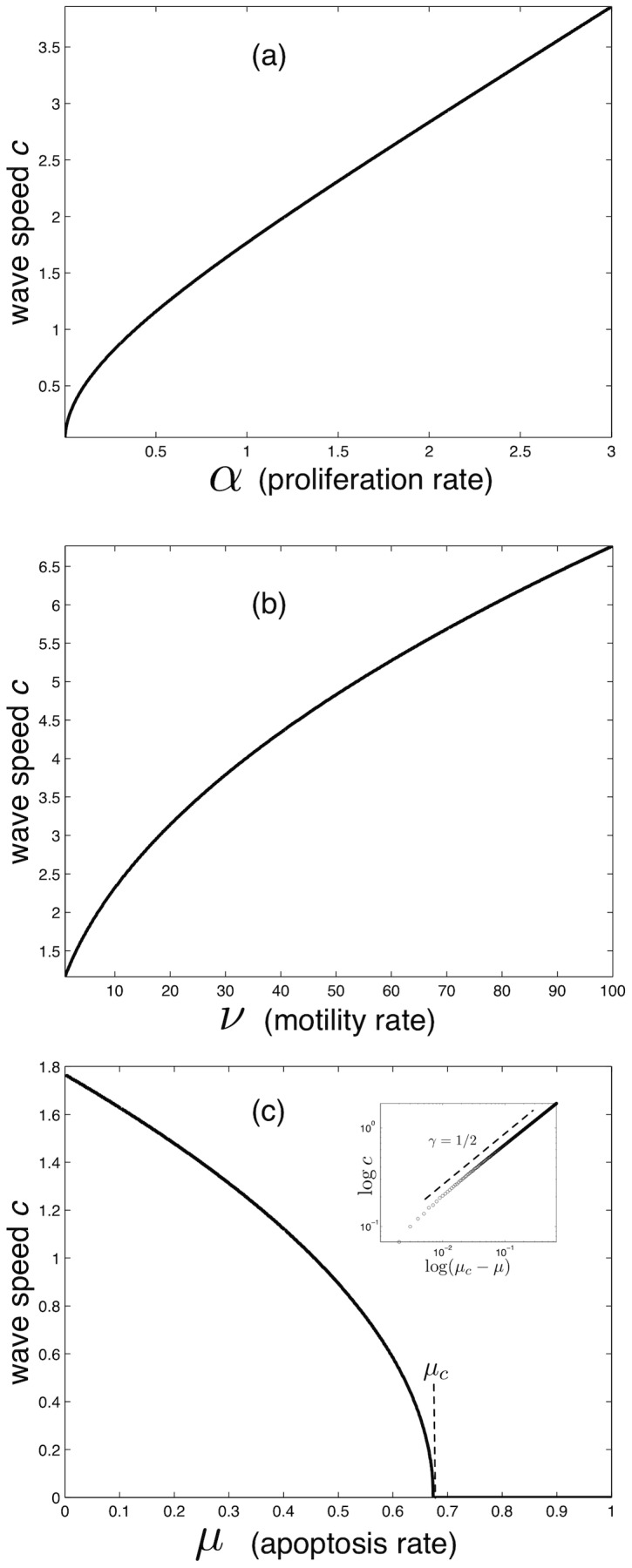
Impact of model parameters. The wave speed of the propagating tumour margin as a function of (a) 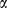, (b) 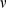 and (c) 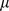. The phenotypic switching rates were fixed at 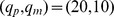. The dashed line in the inset of (c) has slope 1/2 and shows that 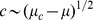.

Naturally the other model parameters also affect the rate of tumour invasion (see figure 7). Increasing the proliferation rate 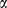 leads initially (for small 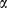) to an increase in velocity according to 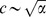, while for 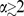 there is a cross-over to a linear dependence with 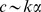, with 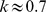. The motility rate 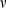 also influences the wave speed in a non-linear way according to the relation 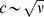, which holds for all 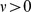. Finally, increasing the rate of apoptosis 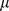, as expected, decreases the wave speed, and does so in a non-linear way. Actually the dependency on 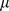 looks very much like that of a second-order phase transition, where the derivative 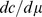 diverges at a critical point 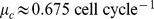, and we have for 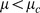 that 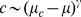 (see inset of figure 7c). We observed that the critical apoptosis rate 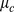, above which no travelling wave solutions exists and hence the tumour disappears, depends on the other parameters of the model, but that the critical exponent 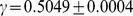 is independent of the other parameters.

## Discussion

### Phenotypic switching affects growth by altering the tumour interface composition

Our model gives considerable insight into the dependency between five cell-level parameters (switching rates 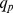 and 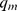, motility rate 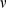, proliferation rate 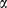 and rate of apoptosis 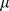) and the macroscopic dynamics of tumour growth and invasion. Focusing on the impact of the phenotypic switching rates we showed that tumour cells with a small 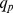 and large 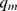 (see fig. 3) give rise to small tumours (low 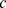) while those characterised by a large 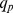 and intermediate 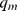 grow into large tumours (high 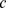). To see why this is the case, consider a one-dimensional growth process in which the tumour expands in a narrow channel. If 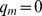, then the tumour expands only through proliferation of the cells at the interface (since interior cells cannot divide), and the interface thus moves with velocity 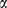, equal to the proliferation rate. If 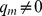 then cells at the interface spend some time in the motile state, freeing up space and allowing previously blocked cells to proliferate. This process increases the interface velocity, but it is also clear that a large 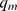 has a negative effect on tumour growth, since if 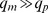 fewer cells are in the proliferative state and can thus take advantage of the space created via cell migration. From this line of reasoning it is clear that the tumour interface velocity will depend on 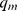 in a non-monotone way. Taken together, our analysis shows that 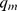 and 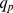 affect glioblastoma progression by altering the composition and structure of the tumour interface, and that for each 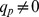 the velocity 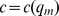 attains a maximum, which occurs at 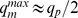.

The above reasoning, and our model, do however not take into account the effects of mechanical forces between the cells. In particular it is, in real tumours, possible for cells to push one another and hence to divide and move, although there is no free space. This process will most likely lessen the positive effect of cell migration on tumour growth, but since it has been experimentally established that few cell divisions occur in the core of the tumour due to pressure build-up and hypoxia, we believe that the conclusions of our model still hold to a large extent.

A similar trade-off between proliferation and migration has in fact been observed in the models of Hatzikirou et al. and Fedotov and Iomin [29]. Although a formal comparison with the former model is difficult, the macroscopic equations that Hatzikirou et al. derive show that the number of rest channels (comparable to the likelihood of a cell proliferating), increases the proliferation rate, but at the same time decreases the motility of the cells. In the work of Fedotov and Iomin [29] a similar trade-off is present. Using a continuous time random walk model they showed that if the waiting times in the P- and M-state are exponentially distributed (as in our model) then the margin velocity is non-monotone in the ratio 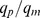 and that the maximum velocity is achieved for 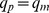. However it should be noted that their model does not take size exclusion into account, and hence yields an overestimate of the effects migration has on invasion.

A trade-off between proliferation and migration has also been investigated in relation to cancer stem cells and tumour progression by Enderling et al. [35]. They showed that cell migration can lead to the formation of secondary tumour loci, in a process termed self-metastasis, which might accelerate tumour growth, depending on the ratio of migratory and proliferative behaviour. In a related study it was shown that cancer stem cell migration might lead to branched tumour morphology and that it can increase the chance of tumour recurrence [36]. These modelling results together with those presented in this study highlight the importance of cell migration in tumour progression and motivate future experimental studies.

### The model recapitulates the wave speed dependency in the Fisher equation

We have also demonstrated that the other parameters in the model affect the speed of invasion. Firstly, the impact of the motility rate and the proliferation rate imply that the wave speed dependence observed in the Fisher equation (1), 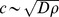, also holds in our system, when equating the diffusion constant 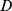 with the motility rate 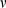 and the proliferation rate 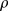 with 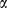 (at least for small and biologically realistic values of 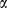). The Fisher equation has been shown to give an accurate macroscopic description of glioblastoma progression *in vivo* [22], which also lends support to our model. The correspondence to the Fisher equation is particularly interesting since it allows for a connection between cell level characteristics (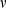 and 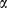) and tissue-scale behaviour, and suggest a means of parametrising our model, not only using single-cell measurements, but also from tissue-level data, such as MRI-scans. From consecutive images the position and hence velocity of the invading tumour margin can be determined and compared with the results of the model.

### Progression depends on apoptosis rate in a discontinuous fashion

Secondly, we observed a second-order phase transition in the velocity with respect to the rate of apoptosis 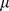. This means that there is a critical apoptosis rate 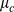 above which no tumour can grow and that for 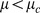 we have 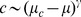, where 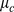 is parameter-dependent, but 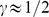 seems to hold for all parameter values. The discontinuous behaviour of 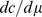 is interesting, not only from a theoretical perspective, but also because it implies that if a high enough rate of apoptosis is induced, it may not only retard tumour growth, but in fact lead to regression. However, these results should viewed with caution, since the model would need to be modified and extended in order to properly account for the dynamics of drug delivery and treatment (cf. [37]).

### Experimental implications of our model

While data from Farin et al. [13] served as the impetus for our model, we note that a few additional experiments support our modeling assumptions. First, the general observation that glioma cells sampled from invasive fast-growing tumours are characterised by a blend of proliferative and migratory behaviour [2] supports our results, although only in a qualitative way. Second, a recent study on different glioma subclones obtained from the same patient identified a particular cell type as being particularly invasive. Subsequent analysis of proliferation of these clones (determined by Ki67-staining) showed that the most invasive subclone (giving rise to the largest tumours *in vivo*) had the lowest proliferation rate [38]. Although the subclones were not subject to a motility assay, these results still diminish the importance of cell proliferation in determining tumour growth rate, and future studies that measure both proliferation and migration could be even more useful in this respect.

In order to gain further experimental support for our model, we plan in future work, to measure the five cell specific parameters directly. Such measurements should be possible by applying live imaging microscopy techniques to primary glioblastoma-derived cell cultures. A first application of such measurements could be exploited to develop the model further, to predict progression for an individual patient based on cell-level phenotyping, and to develop chemical compound screens where the impact of a chemical on the model parameters are observed. This might in turn lead to a strategy to define *in vivo*-relevant compounds more likely to inhibit progression.

### Future work

The current model is however far from these highly set goals, and there are a number of extensions that would make the model more realistic. In its current form the model does not account for cell-cell adhesion, which could be incorporated letting the motility rate 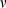 be dependent on the neighbourhood of the cell [25], [26]. The preferential migration along capillaries and myelin tracts, and the tendency for glioma cells to divide at capillary branch points, is also something that could be included. A further complication is that cancer cells within a real glioblastoma are not identical with respect to their behaviour, but exhibit both genotypic and phenotypic heterogeneity, e.g. cells with a migratory phenotype tend to be located at the tumour boundary whereas dividing cells are commonly found in the main tumour mass, a fact which is not captured by the current model.

Despite this we would still expect the results of our model to hold at least with respect to the large-scale behaviour of the tumour. The real situation is also complicated by the fact that cancer cells are selected for based on their phenotype. One hypothesis which emerges from our model is that selection could drive the behaviour of the cells to the optimal balance between 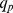 and 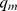, although this hypothesis would require a model that allows for population heterogeneity in order to be tested.

Adding these mechanisms would of course make the model less tractable from an analytical point of view, but this trade-off between simplicity and reality is something that all modellers must deal with.

## Methods

### Derivation of continuum model

Let us consider a one-dimensional lattice indexed by the integers. We let 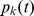 denote the probability of finding a P-cell at site 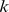 at time 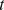, and equivalently let 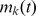 represent the occupation probability of M-cells. The general strategy is to formulate two coupled master equations for the occupation probabilities, which will then be approximated by a set of PDEs, amenable to a wave speed analysis that hopefully will reveal the influence of 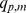 on tumour growth.

Let us first consider 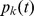. Which are the processes that affect this quantity at a given site?

1. an existing P-cell can die through apoptosis (with rate 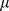)
2. an existing P-cell might switch to an M-cell (with rate 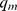)
3. an M-cell residing at the site might switch into becoming a P-cell (with rate 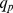)
4. a neighbouring cell might divide placing its offspring in the (empty) considered site (with rate 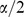)

Summarising all these processes we can write:
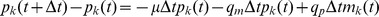


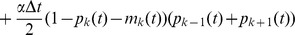
where the first term is a loss term due to apoptosis, while the second and third term are due to phenotypic switching. The final term is due to cell division from the neighbouring sites, and here we have made use of the independence assumption discussed above. After dividing both sides by 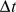 and going to the limit 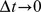 we end up with the following expression:
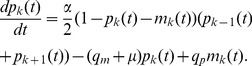
(5)


In order to simplify the expression and also draw parallels to continuum systems we define a discrete Laplace operator
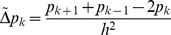
(6)where 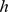 corresponds to the spacing of the lattice. Equation (5) can now be written as
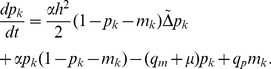
(7)


If we now turn to the motile cells, the following processes affect 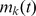:

1. an existing M-cell can die through apoptosis (with rate 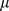)
2. an existing M-cell might switch to a P-cell (with rate 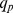)
3. a P-cell residing at the site might switch into becoming a M-cell (with rate 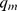)
4. an existing cell might move away from the considered site (with rate 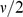 in each direction)
5. a neighbouring cell might move into the (empty) considered site (with rate 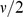)

Taking all these processes into account we can write
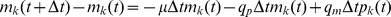


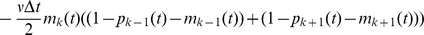


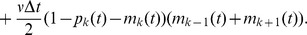



The first three terms can be recognised as apoptosis and switching terms, while the fourth and fifth are due to movement out of and into the site. After a bit of algebra and making use of the discrete Laplacian defined in eq. (6) we get
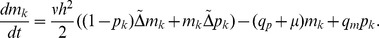



In summary we have that the time evolution of the occupation probabilities are described by the following coupled equations:
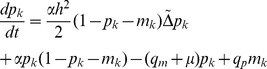
(8)

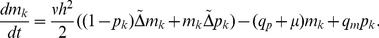
(9)


Please note that despite the similarity to PDEs, that describe the changes of a quantity in continuous space and time, these equations are defined on the lattice and describe the probability of finding a cell of a specific type in a certain location. In many instances it is natural to proceed by taking the spatial continuum limit of the discrete master equation(s), but in this case, where we are considering expansion via both cell movement and pure cell division (the case 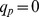), things are a bit more delicate, because when the size of the cells tend to zero (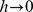) so does the contribution of cell division to tumour expansion. In order to achieve a sensible continuum limit, a certain scaling in space and time is required, which implicitly assumes that cell motility occurs on a much faster time scale than cell division [39], something which is generally not the case in the case of glioma biology.

However in order to proceed with the analysis and make use of the toolbox of real analysis we will approximate the above equations with the following PDEs:
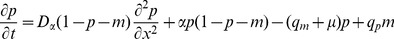
(10)

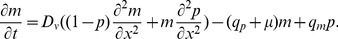
(11)


The motivation behind this choice is that the master equations (8) and (9) are the (space) discretised versions of (10) and (11). The diffusion constants are given by 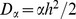 and 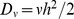, where 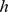 is the spacing of the lattice, which we for simplicity measure in terms of cell size, and accordingly set 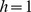. This means that we consider the dynamics on the length scale of a single cell. Please note that the unit of the diffusion constants 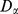 and 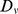 is 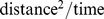, while the unit of the underlying proliferation and migration rates is 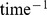. The correspondence between the master equations and PDEs is, however, not rigorous and implies that the analytic results obtained for the PDEs are not in general valid for the master equations, but, as we shall see, still reflect the behaviour of the IB-model to a large extent.

### Phase-space analysis

For the sake of clarity let us recapitulate the method applied to the Fisher equation (1) in order to calculate its speed of invasion. The travelling wave ansatz (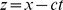) turns the Fisher equation into second order ODE in the variable 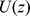. By introducing the variable 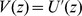 the ODE is turned into a two-dimensional autonomous system. The system has two fixed points 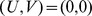 and 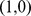, and by calculating the eigenvalues of the Jacobian (a determinant of partial derivatives) at the two fixed points, one finds that the fixed point at 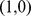 (corresponding to the invaded state) is a saddle point (independent of 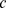), while the characteristics of the one at the origin depend on 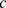. For 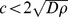 the fixed point is a stable spiral, while for 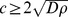 it is a stable node. The heteroclinic orbit connecting the two states goes from (1,0) through the third quadrant (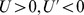, as in figure 4), and only if the origin is a stable node does it enter the fixed point without spiralling around and attaining negative values of the density 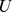 of cancer cells. Negativity would be inconsistent with the non-negative solution of the equation (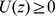), and shows that the smallest possible wave speed is given by 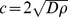.

In our case the travelling wave ansatz transforms the system of PDEs (10)–(11) to the following set of coupled ordinary differential equations (ODE):
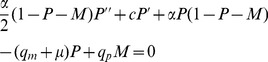
(12)

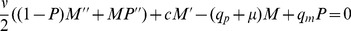
(13)where prime indicates derivative with respect to 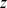, and where we have expressed the diffusion coefficients in terms of 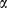 and 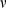. Because 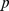 and 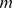 represent occupation probabilities we seek solutions 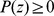 and 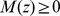 for all 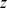. In order to perform a phase-space analysis we need to transform the coupled ODEs to an autonomous system by introducing the variables 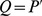 and 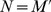. This expands equation (12) and (13) into the following four-dimensional system:
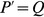
(14)

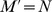


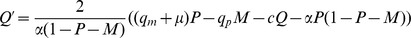


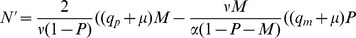


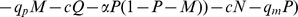
with boundary conditions
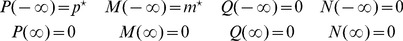
(15)where
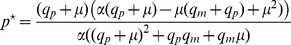
(16)and
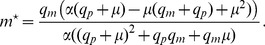
(17)


The boundary conditions reflect the fixed points of the system, which are 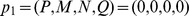 and 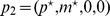, and correspond to the healthy and invaded state respectively. In the limit 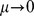 the invaded fixed point simplifies to 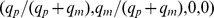, in which case only the relative magnitude of the switching rates 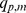 determines the equilibrium occupation probabilities (cf. the values of 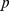 and 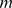 at 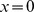 in figure 4b and c).

What will help us determine the wave speed 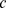 is the characteristics of these fixed points, or more precisely the one at the origin. This method only gives a lower bound on the wave speed, but this minimal 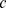 turns out to be the one attained for relevant initial conditions for the Fisher equation [21], although this requires further proof [40].

We will now apply the same kind of reasoning of non-negativity as for the Fisher equation in order to obtain a minimal wave speed 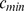 for our system (3)–(4). The properties of the fixed point at the origin are determined by linear stability analysis and depend on the eigenvalues of the Jacobian 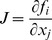, where the 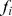
's are the right hand sides of equation (14) and 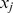 correspond to the independent variables 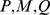 and 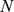. The Jacobian evaluated at the origin is given by
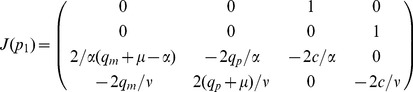
(18)whose eigenvalues are given by the zeros of the characteristic polynomial
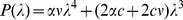
(19)

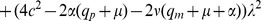


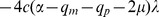


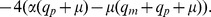



The roots of this equation 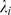 have, for the biologically relevant parameter values and 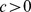 non-zero real part, 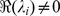, implying that the fixed point is hyperbolic and thus that its characteristics are fully determined by linear stability analysis [41]. The aim is now to find the smallest 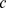 such that all roots of 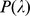 have imaginary part equal to zero, since only then are we guaranteed trajectories which do not oscillate around the origin, and remain positive in the variables 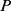 and 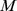, which is required since these variables represent non-negative occupation probabilities. Determining the smallest such 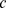 turns out to be intractable from an analytic point of view, and we will therefore resort to numerical solution of the eigenvalue problem.
